# The *insulin-like growth factor 2* gene in mammals: Organizational complexity within a conserved locus

**DOI:** 10.1371/journal.pone.0219155

**Published:** 2019-06-28

**Authors:** Kabita Baral, Peter Rotwein

**Affiliations:** 1 Graduate School, College of Science, University of Texas at El Paso, El Paso, Texas; 2 Department of Molecular and Translational Medicine, Paul L. Foster School of Medicine, Texas Tech Health University Health Sciences Center, El Paso, Texas; University of Münster, GERMANY

## Abstract

The secreted protein, insulin-like growth factor 2 (IGF2), plays a central role in fetal and prenatal growth and development, and is regulated at the genetic level by parental imprinting, being expressed predominantly from the paternally derived chromosome in mice and humans. Here, *IGF2/Igf2* and its locus has been examined in 19 mammals from 13 orders spanning ~166 million years of evolutionary development. By using human or mouse DNA segments as queries in genome analyses, and by assessing gene expression using RNA-sequencing libraries, more complexity was identified within *IGF2/Igf2* than was annotated previously. Multiple potential 5’ non-coding exons were mapped in most mammals and are presumably linked to distinct *IGF2/Igf2* promoters, as shown for several species by interrogating RNA-sequencing libraries. DNA similarity was highest in *IGF2/Igf2* coding exons; yet, even though the mature IGF2 protein was conserved, versions of 67 or 70 residues are produced secondary to species-specific maintenance of alternative RNA splicing at a variable intron-exon junction. Adjacent *H19* was more divergent than *IGF2/Igf2*, as expected in a gene for a noncoding RNA, and was identified in only 10/19 species. These results show that common features, including those defining *IGF2/Igf2* coding and several non-coding exons, were likely present at the onset of the mammalian radiation, but that others, such as a putative imprinting control region 5’ to *H19* and potential enhancer elements 3’ to *H19*, diversified with speciation. This study also demonstrates that careful analysis of genomic and gene expression repositories can provide new insights into gene structure and regulation.

## Introduction

Insulin-like growth factor 2 (IGF2), a 67-amino acid single-chain secreted protein, plays a central role in human fetal growth and development, and is involved in a variety of physiological and patho-physiological processes in other mammalian species [1–6]. Over-expression of IGF2 in humans appears to be responsible for the asymmetric organ and tissue overgrowth observed in Beckwith-Wiedemann syndrome [7, 8], and its diminished expression appears to cause the reduced growth and bodily dysmorphism seen in Silver-Russell syndrome [7, 8]. A single nucleotide polymorphism in a transcriptional repressor binding site in an *IGF2* gene promoter alters promoter activity and levels of IGF2 in skeletal muscle, and thus controls muscle mass in pigs [9, 10], and possibly in other mammals [11], while in mice, targeted *Igf2* gene knockout causes reduced fetal growth [12].

Human *IGF2* and mouse *Igf2* genes each reside within a conserved linkage group on human chromosome 11p15.5 and mouse chromosome 7, respectively. The locus also includes tyrosine hydroxylase (*TH*/*Th*), *INS* (*Ins2* in mice), *H19*, and other genes. In both species, parental imprinting reciprocally regulates expression of *IGF2/Igf2* and *H19* genes in most cells and tissues [13, 14]. *IGF2/Igf2* is active on the paternally derived chromosome, and *H19* on the maternal chromosome [13, 14]. An imprinting control region (ICR) mediates this chromosome-of origin-specific gene expression via DNA sequences that encode recognition sites for the protein, CCTC binding factor (CTCF) [15–18]. CTCF binds to the ICR in maternal chromatin, and thereby directs distal enhancers to the *H19* promoter while simultaneously blocking their access to *IGF2/Igf2* promoters [16, 17, 19]. In paternal chromatin ICR DNA becomes methylated on cytosine residues in CpG dinucleotides, which interferes with CTCF binding, and thus allows the enhancers to activate *IGF2/Igf2* [16, 17, 19].

Human *IGF2* and mouse *Igf2* genes each have complicated structures and patterns of gene expression [12, 13, 20, 21]. The human *IGF2* gene contains 10 exons and 5 promoters [13, 14, 21, 22], while mouse *Igf2* contains 8 exons and 4 promoters [23–25]. Human *IGF2* gene expression and protein biosynthesis continues throughout life [21, 26], but in mice it vanishes in most tissues within a few weeks after birth [12, 13, 20]. It thus had been postulated that the extra human promoter was responsible for life-long *IGF2* gene activity [27]. This idea now appears to be incorrect, as recent data show that several *IGF2* gene promoters, including those with mouse homologues, are active in multiple adult human tissues [28]. Thus, the molecular mechanisms responsible for maintaining or limiting IGF2 protein production during the lifespan in different species have not yet been delineated.

Recent advances in genomics present new opportunities for gaining insights into genetic determinants of physiology, disease predisposition, and evolution [29–31] through comparative analysis of genomic information [32]. The present studies were initiated as a means of gaining insight into key aspects of *IGF2/Igf2* gene and *IGF2/Igf2*—*H19* locus structure and regulation as they have evolved during mammalian speciation. Using data extracted from public repositories, *IGF2/Igf2*—*H19* loci, genes, and gene expression patterns were analyzed in 19 mammalian species representing 13 orders and spanning ~166 million years (Myr) of evolutionary diversification [33–36]. The results demonstrate extensive conservation in coding regions of *IGF2/Igf2* exons and in IGF2 proteins, the presence of several moderately conserved 5’ untranslated (UTR) exons in *IGF2/Igf2*, along with data supporting the use of multiple promoters in many species, and divergence in both *H19* gene structure and locus enhancers and boundary elements. Thus, it appears that some common paradigms governing *IGF2/Igf2* gene regulation and IGF2 functions were present at the onset of mammalian diversification, but that other locus features developed during further speciation.

## Materials and methods

### Genome database searches and analyses

Mammalian genomic databases were accessed in the Ensembl Genome Browser (www.ensembl.org) and the UCSC Genome Browser (https://genome.ucsc.edu). Searches were performed with BlastN under normal sensitivity (maximum e-value of 10; mis-match scores: 1,-3; gap penalties: opening 5, extension, 2; filtered low complexity regions, and repeat sequences masked) using as queries human *IGF2* or *H19* DNA segments or other nearby genomic regions (*Homo sapiens* genome assembly GRCh38.p12), or mouse *Igf2* or *H19* gene DNA segments, and adjacent regions (*Mus musculus*, genome assembly GRCm38.p6). The following genome assemblies were queried: armadillo (*Dasypus novemcinctus*, Dasnov3.0), cat (*Felis catus*, Felis_catus_9.0), cow (*Bos taurus*, ARS-UCD1.2), dog (*Canis lupus familiaris*, CanFam3.1), elephant (*Loxodonta africana*, LoxAfr3.0), gorilla (*Gorilla gorilla*, gorGor4), guinea pig (*Cavia porcellus*, cavpor3.0), horse (*Equus caballus*, EquCab3.0), megabat (*Pteropus vampyrus*, pteVam1), olive baboon (*Papio anubis*, Panu_3.0), opossum (*Monodelphis domestica*, monDom5), pig (*Sus scrofa*, Sscrofa11.1), platypus (*Ornithorhynchus anatinus*, OANA5), rabbit (*Oryctolagus cuniculus*, OryCun2.0), rat (*Rattus norvegicus*, Rnor_6.0), Tasmanian devil (*Sarcophilus harrisii*, Devil_ref v7.0), and wallaby (*Macropus eugenii*, Meug_1.0). Additional searches were conducted using as queries other mammalian cDNA and genomic sequences to follow-up, verify, or extend initial results. For example, portions of koala *Ins* (obtained from genome assembly for *Phascolarctos cinereus*, phaCin_tgac_v2.0) were used to search the Tasmanian devil genome. Mammalian *IGF2/Igf2* and H19 cDNAs were obtained from the National Center for Biotechnology Information (NCBI) nucleotide database for cat, cow, dog, elephant, guinea pig, horse, opossum, pig, platypus, rabbit, Tasmanian devil, and wallaby. Other conserved DNA sequences were identified using the ECR (evolutionarily conserved regions) browser (https://ecrbrowser.dcode.org/). Sources for IGF2 protein sequences included the Uniprot browser (http://www.uniprot.org/), GENCODE/Ensemble databases, and the NCBI Consensus CDS Protein Set (https://www.ncbi.nlm.nih.gov/CCDS/). When primary protein data were unavailable, for example, for megabat and wallaby, DNA sequences from *IGF2/Igf2* exons were translated with assistance of Serial Cloner 2.6 (see: http://serialbasics.free.fr/Serial_Cloner.html).

### Protein alignments

Multiple sequence alignments were performed for the mature IGF2 protein, IGF2 signal peptides, and E domains. Amino acid sequences were uploaded into the command line of Clustalw2 (https://www.ebi.ac.uk/Tools/msa/clustalw2/), the latest version of Clustal, in FASTA format. This program first performs pairwise sequence alignments using a progressive alignment approach, after which it creates a guide tree using a neighbor joining algorithm, which is then used to complete a multiple sequence alignment. The output files were in GCG MSF (Genetics Computer Group multiple sequence file) format.

### Analysis of *IGF2/Igf2* and *H19* gene expression

Examination of *IGF2/Igf2* or *H19* gene expression in different mammals was conducted using the NCBI Sequence Read Archive (NCBI SRA) (www.ncbi.nlm.nih.gov/sra), using the individual RNA sequencing libraries listed in S1 Table. Searches were performed with 60-nucleotide DNA segments comprising (a) 30-nucleotides from the 3’ end of mammalian equivalents of human *IGF2* exons 2, 3, 4, 5, 6, or 7, which was joined to 30-nucleotides from the 5’ end of the equivalent of human *IGF2* exon 8 (the most 5’ coding exon), or (b) 30-nucleotides from the 3’ end of mammalian equivalents of human *IGF2* exon 8 fused to the 30-nucleotides from the 5’ end of the equivalent of exon 9 (the first two coding exons). Similar searches used 60-nucleotides from the mammalian equivalents of human *H19* exons 1, 2 or 4, and 60-nucleotides from the mammalian equivalents of human *MRPS17* exon 3, the latter being a presumptively constitutively expressed control gene (see S2 Table for DNA sequences). All queries used the Megablast option (optimized for highly similar sequences; maximum target sequences–10,000 (this parameter may be set from 50 to 20,000); expect threshold–10; word size–11; match/mismatch scores–2, -3; gap costs–existence 5, extension 2; low-complexity regions filtered).

Data are presented in text and Tables as percent identity over the entire query region, unless specified otherwise.

## Results

### The mouse *Igf2*—*H19* and the human *IGF2*—*H19* loci and genes

**Fig 1:**
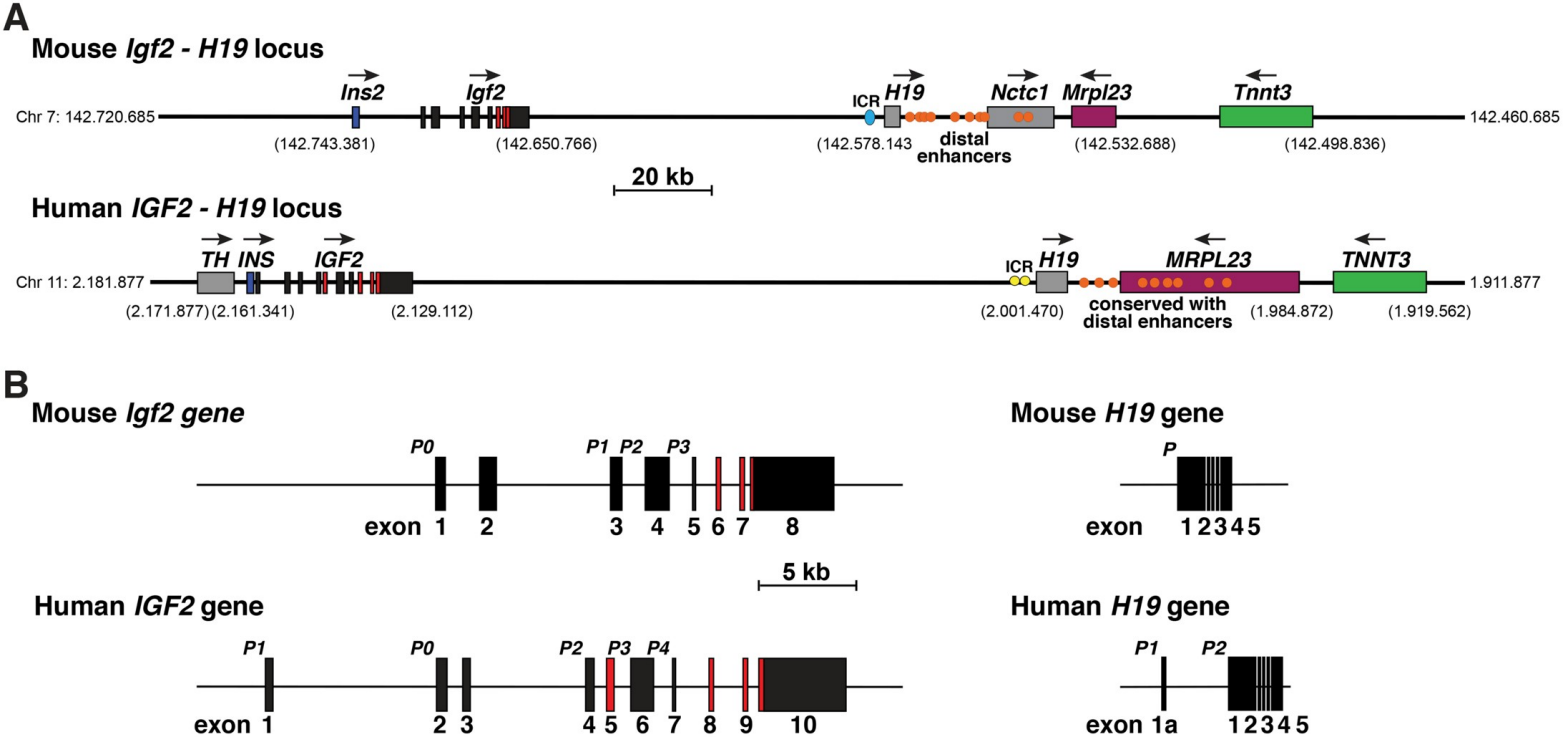
The mouse *Igf2—H19* locus and the human *IGF2—H19* locus and genes. **A**. Maps of the mouse *Igf2 –H19* locus on chromosome 7 and the human *IGF2 –H19* locus on chromosome 11p15.5 with chromosomal coordinates for key features listed. For *IGF2/Igf2*, exons are illustrated as boxes, with coding regions in red and noncoding in black, and introns as horizontal lines. Other genes are depicted as single boxes and include tyrosine hydroxylase (*TH*), insulin (*Ins2*, *INS*), noncoding RNA genes *H19* and *Nctc1*, mitochondrial ribosomal protein L23 (*Mrpl23*, *MRPL23*), and troponin T3, fast skeletal type (*Tnnt3*, *TNNT3*). Horizontal arrows show the direction of gene transcription. Blue or yellow circles represent the mouse or human imprinting control region (ICR), respectively, which are located 5’ to *H19* [15–17]; orange ovals indicate the 10 distal enhancers that were identified and functionally mapped in the mouse genome [40], and their 9 human homologues ([37] labeled as ‘conserved with distal enhancers’). A scale bar is indicated. **B**. Detailed view of mouse *Igf2* and human *IGF2* and both *H19* genes, with exons as boxes (8 for *Igf2*, 10 for *IGF2*, 5 for mouse *H19*, and 6 for human *H19*), and introns and flanking DNA as horizontal lines. The letter ‘P’ indicates gene promoters (P0 and P1 –P3 for *Igf2*, P0 and P1 –P4 for *IGF2*, P for mouse *H19*, and P1 and P2 for human *H19*), and a scale bar is shown. For *Igf2* and *IGF2*, non-coding exons are in black and coding exons are colored red. For *H19*, all exons are in black.

The mouse *Igf2*—*H19* locus on chromosome 7 and the human *IGF2—H19* locus on chromosome 11p15.5 each encode the same 5 protein-coding genes (*Th*/*TH*, *Ins2*/*INS*, *IGF2/Igf2*, *Mrpl23*/*MRPL23*, and *Tnnt3*/*TNNT3*), along with several genes expressing non-coding RNAs, of which the most well-known is *H19* [14, 37] (Fig 1A). As noted in the Introduction, *IGF2/Igf2* and *H19* gene activity in both species is influenced by parental imprinting, with H19 mRNA being expressed from the maternally derived chromosome, and *IGF2/Igf2* from the paternal chromosome through differential access to distal enhancers found 3’ to *H19* [15–18, 38, 39]. At least 10 of these enhancer elements have been mapped in the mouse genome 3’ to *H19* on chromosome 7, and have been examined functionally in transgenic mice for enhancer properties [40] (Fig 1A). Of note, the first 7 elements, CS1 –CS7, are located in intergenic DNA, and the last 3 (CS8 to CS10) either just 5’ to *Nctc*1 or in *Nctc1* intron 2 (Fig 1A, [41, 42]). DNA similarity searches revealed sequences corresponding to 9 of these 10 segments in relatively analogous locations on human chromosome 11p15.5 (Fig 1A), although nucleotide identity was fairly limited [28, 37], and no studies have been performed to validate their possible functions. Five of the 9 human elements map within the *MRPL23* gene. CS6 -CS8 are found in intron 5, and CS9 and CS10 in intron 4, while CS5 overlaps the exon 5 –intron 5 junction (Fig 1A).

### The *IGF2/Igf2* gene in mammals

**Fig 2:**
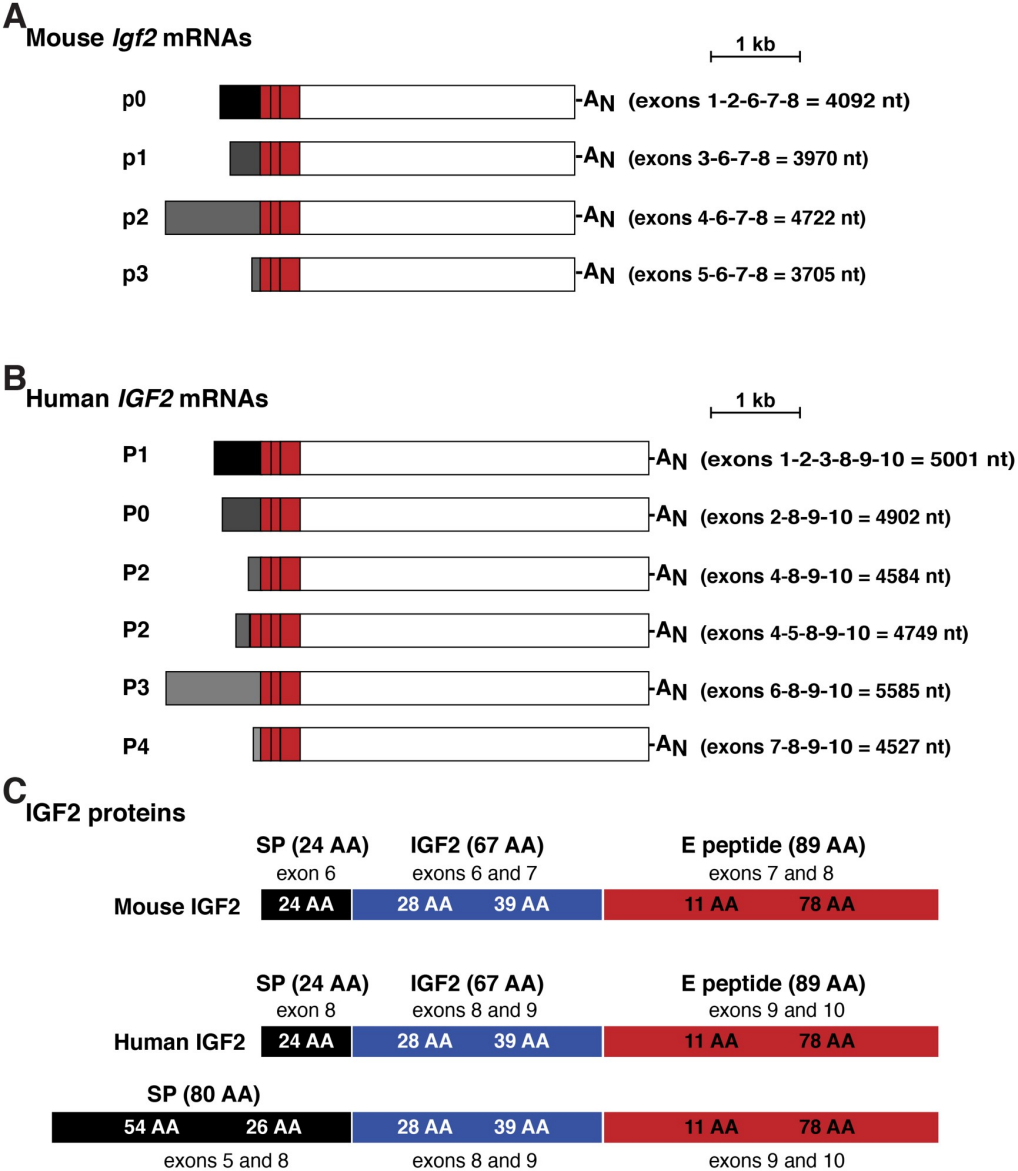
Mouse *Igf2* and human *IGF2* mRNAs and proteins. **A**, **B**. Depiction of the 4 major species of mouse *Igf2* transcripts (A) and the 6 major types of human *IGF2* mRNAs (B). The responsible promoters (p for mouse, P for human) are listed, as are the exons found in each transcript. The length of each mRNA is in nucleotides (nt). A_N_ represents the polyadenylic acid tail found at the 3’ end of mRNAs. **C**. Depiction of mouse and human IGF2 protein precursors, showing the derivation of each segment from different *Igf2* or *IGF2* exons. Mature, 67-amino acid IGF2 is in blue; presumed and confirmed signal peptides (SP) are in black, and the 89-amino acid E peptide is in red. Amino acid is abbreviated as AA.

Based on primary peer-reviewed publications and analysis of Ensembl and UCSC Genome Browsers, mouse *Igf2* comprises 8 exons, with gene transcription being controlled by 3 adjacent promoters, p1—p3, and a more 5’ promoter, p0, each with a distinctive non-coding 5’ leader exon or exons, while exons 6–8 encode the IGF2 precursor protein [23–25] (Figs 1B and 2A). Human *IGF2* by contrast has 10 exons and 5 promoters, including an additional upstream promoter and associated noncoding exon, and a fourth alternatively expressed coding exon (exon 5, Figs 1B and 2A) [13, 14, 21, 22]. Only 8 of the 10 exons are found in *IGF2* transcripts in adults according to the Genotype-Tissue Expression Project (GTEX release 7) [37], which has collected data on many human tissues by RNA-sequencing [43, 44]. As in the mouse, the 5 human *IGF2* promoters each control expression of distinctive non-coding exons, but all include exons 8–10 that encode the IGF2 protein precursor and 3’ un-translated RNA (Fig 2B). The main differences between mouse and human *IGF2/Igf2* are human promoters 1 and 2 (P1 and P2). P1 is distinctly human, while P2 regulates two classes of *IGF2* transcripts that differ by alternative splicing of exon 5. Inclusion of exon 5 in a cohort of human *IGF2* mRNAs leads to an alternative predicted IGF2 precursor protein of 236 amino acids, including an 80-residue NH_2_-terminus that is lacking in the mouse (Fig 2C).

**Fig 3:**
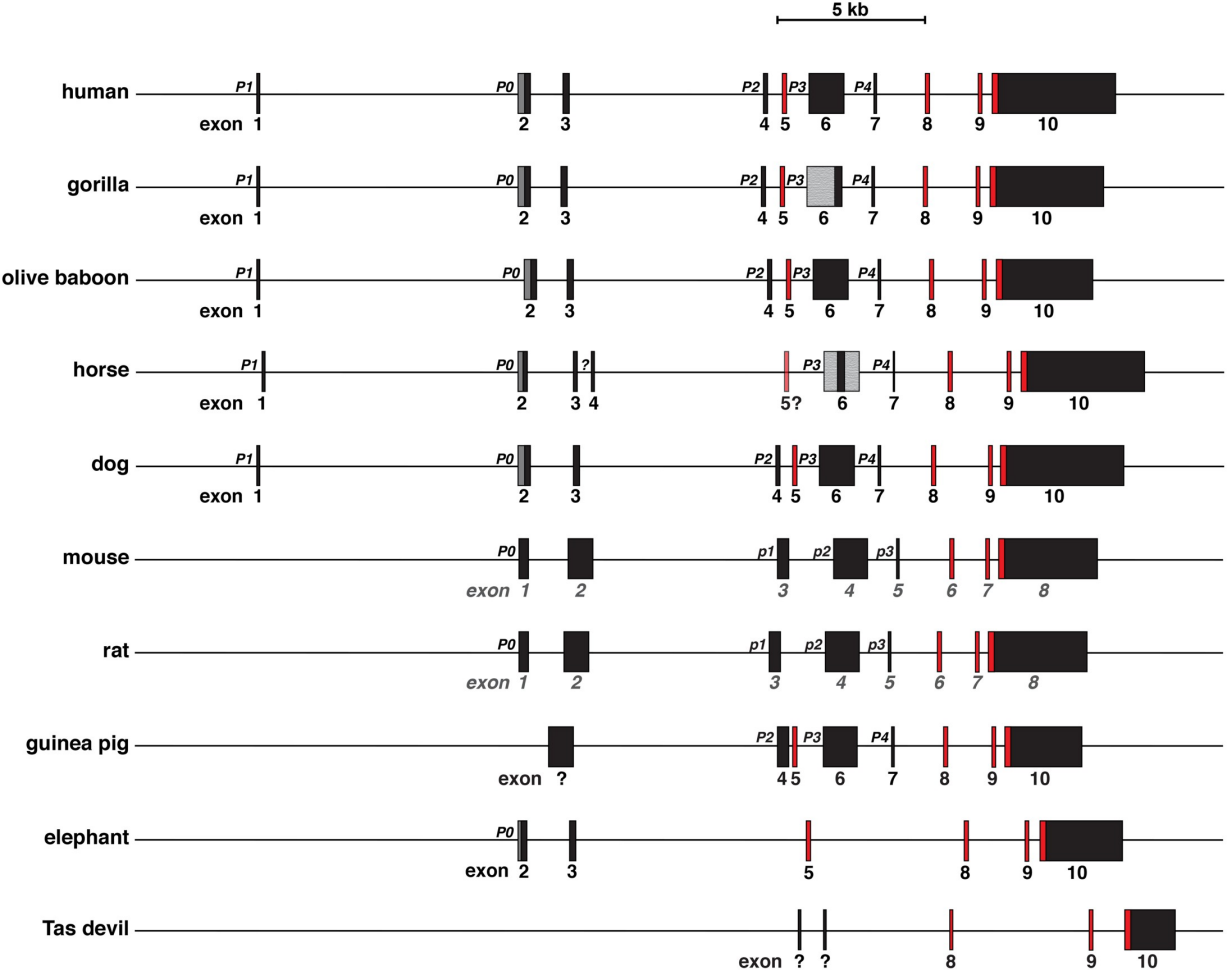
Comparison of *IGF2/Igf2* genes among mammals. Schematics of human, gorilla, olive baboon, horse, and dog *IGF2*, and mouse, rat, guinea pig, elephant, and Tasmanian (Tas) devil *Igf2* genes are shown. These are the genes for which the most information was extracted by searching genomic databases, as described in ‘Materials and methods’. Promoters (P) are labeled; the different terminology employed for mouse and rat promoters p1 to p3 (lower case) derives from genomic databases. All exons are indicated as boxes, with non-coding exons in black or gray and coding exons in red. The dark gray regions in exon 2 in human, gorilla, olive baboon, horse, and dog genes represent the additional part of the exon that is transcribed when P0 is active (exon 2 lg [large] in Table 1). Only the smaller black segment of exon 2 is transcribed when P1 is active (exon 2 in Table 1); it results from exon 1 splicing into exon 2 (see Fig 2B). The lighter gray portions of gorilla and horse exon 6 depict areas that have not been characterized because of poor quality DNA sequences (see Table 1). A question mark under horse *IGF2* exon 5 indicates that the DNA sequence is found in a cDNA but could not be mapped to the horse genome, most likely because of poor quality genomic DNA sequence. The question mark adjacent to horse exon 4 indicates that no DNA sequence similar to human P2 could be mapped. Question marks under two Tasmanian devil *Igf2* exons signify genomic DNA segments matching two *Igf2* cDNAs that are not similar to *IGF2/Igf2* noncoding exons in other mammalian species. A scale bar is also shown.

By using as queries human *IGF2* and mouse *Igf2* exons and promoter segments, and cDNAs from different mammalian species, *IGF2* also appears to be a 10-exon gene in several non-human primates (Fig 3, Table 1), including a pro-simian, mouse lemur, in which both coding and noncoding exons are highly conserved with human *IGF2* [28, 37], in horse and dog (Fig 3, Table 1), and in cow and pig (Table 1). In nearly all of the species examined, the annotated data were incomplete, even though as described below we were able to identify additional potential exons in the respective genomic databases (e.g., 7 exons characterized in Ensembl and in the UCSC browser in dog, 4 exons in horse and guinea pig, 3 exons in elephant (where the *IGF2* gene is named *PTHR11454 SF10*)). When all of our newly identified and mapped information was considered, there was extensive structural similarity with human *IGF2* gene in gorilla, olive baboon (and several other primates [28, 37]), cow, pig, horse, and dog, and congruence between mouse and rat *Igf2* genes (Fig 3, Table 1). In 7 of 10 other mammals (or a total of 15 of the 18-nonhuman species surveyed here), coding exons equivalent to human exons 8–10 (or mouse exons 6–8) could be identified (Table 1; the outliers here were rabbit, opossum, and platypus, in which no similarities could be detected with other mammals. These exceptions are likely to be secondary to poor genome sequence quality in these 3 species). The equivalents of human or mouse 5’ UTR exons also were found in a variable number of species (i.e., gorilla, olive baboon, cat, and dog for human exon 1; 11 species for exon 2-large, and exons 4, 6, and 7; 10 species for exons 2 and 3; Table 1). Moreover, in several mammals, 5’ UTR exons were identified based on mapping with species-specific *IGF2/Igf2* cDNAs, but the genomic DNA sequences were not sufficiently similar to human or mouse regions to be recognized by BLASTN searches (e.g., cow and pig exons 1 and 3, horse exons 1, 4, and 5; Table 1).

**Table 1:** Percent nucleotide identity with human *IGF2* exons^¶^.

Species	Exon 1 (115 bp)	Exon 2 (220 bp)	Exon 2 lg (478 bp)	Exon 3 (242 bp)	Exon 4 (160 bp)	Exon 5 (165 bp)	Exon 6 (1161 bp)	Exon 7 (103 bp)	Exon 8 (163 bp)	Exon 9 (149 bp)	Exon 10 (4112 bp)
gorilla	99	99	99	99	98	99	98* (229)	100	100	100	97 (3789)
olive baboon	97	95 (204)	94	90	96 (156)	96	98	97	99	99	92 (3514)
cow	No match#	86 (43)	94 (94)	No match#	97 (94)	90 (157)	88 (1146)	86 (91)	94 (138)	89 (123)	85 (659)
pig	No match#	84 (218)	83 (453)	No match#	94 (89)	91 (157)	89	91 (100)	91	91	85 (1029)
horse	No match#	85 (162)	84 (362)	81 (149)	No match#	No match#	86 (227)*	96 (28)#	86 (109)	93	83 (1619)
cat	100 (108)	87 (204)	83 (446)	85 (89)	90 (154)	No match	No match	91 (103)	96 (150)	93 (122)	85 (1593)
dog	82 (117)	89 (204)	86 (342)	93 (44)	90 (137)	85 (157)	88	89 (74)	95 (149)	94 (122)	84 (1466)
mouse	No match	86 (166)	86 (166)	91 (45)	91 (85)	No match	86 (1033)	100 (28)	88 (161)	89	87 (865)
rat	No match	87 (179)	87 (179)	91 (45)	91 (85)	No match	86 (1031)	96 (28)	89	89	86 (767)
guinea pig	No match	No match	No match#	No match	91 (96)	90	89 (1042)	83 (70)	96	95 (127)	84 (969)
rabbit	No match	No match	No match	No match	No match	No match	No match	No match	No match	No match	No match
elephant	No match	87 (203)	84 (339)	88 (83)	No match	91 (91)	No match	No match	90	84 (93)	85 (487)
armadillo	No match	No match	No match	89 (45)	89 (96)	85	89 (376)	91 (44)	91 (130)	94 (110)	84 (516)
megabat	No match	No match	96 (25)	85 (59)	90 (119)	90 (104)	91 (269)*	No match	98 (116)	88	84 (1013)
wallaby	No match	No match	No match	No match	No match	No match	No match	No match	88 (116)	89 (92)	82 (168)
Tas devil	No match	No match	No match#	No match#	No match	No match	No match	No match	93 (120)	84 (81)	86 (102)
opossum	No match	No match	No match	No match	No match	No match	No match	No match	No match	No match	No match
platypus	No match	No match	No match	No match	No match	No match	No match	No match	No match	No match	No match#

^¶^Number of base pairs aligned is in parenthesis if less than length of human exon.

*poor-quality DNA sequence

No match—no DNA sequence identity detected

^#^Exon is present in genome based on match with species-specific DNA.

DNA sequence identity with human *IGF2* exons was highest in coding segments, and ranged from 86–100% for exon 8, 84–100% for exon 9, and 83–97% for exon 10, although in the latter case, the extent of similarity was far less within the 3’ UTR than in coding DNA (Table 1). Untranslated exons generally showed lower levels of identity over smaller regions of the exons than did coding exons (Table 1).

### The *H19* gene in mammals

**Fig 4:**
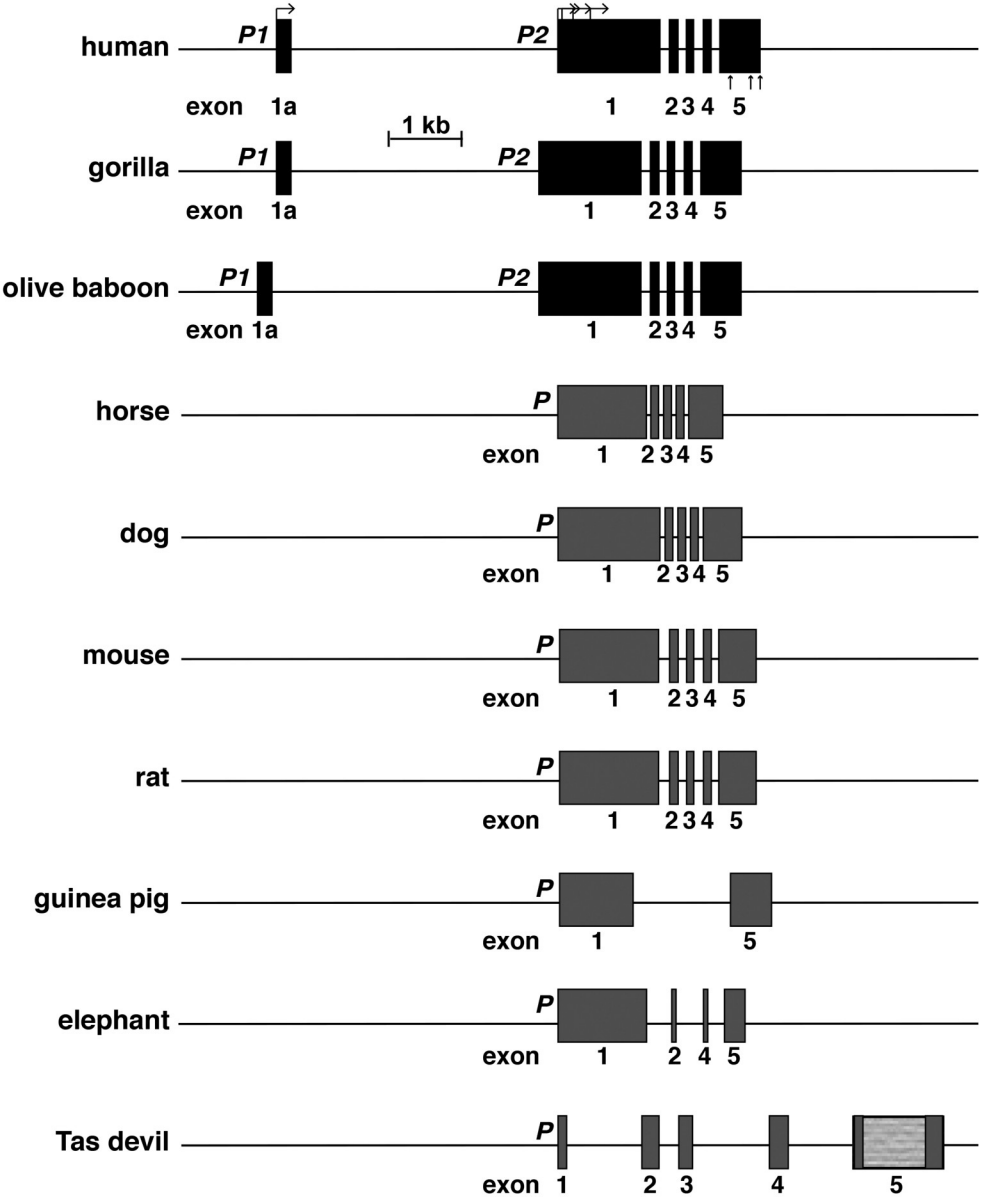
Mammalian *H19* genes. Detailed views of 10 mammalian *H19* genes for which genomic data are relatively complete; exons are boxes, and introns and flanking DNA are horizontal lines. P1 and P2 depict the two gene promoters found in several primates. P denotes the gene promoter in other species. Bent arrows indicate different transcription start sites directed by human P2 and straight vertical arrows depict locations of alternative polyadenylation sites. The Tasmanian (Tas) devil *H19* gene was mapped by similarity with the wallaby gene; the lighter gray portion of Tasmanian devil exon 5 depicts an area that could not be characterized because of poor quality DNA sequence (see Table 2). A scale bar is shown.

Human *H19* is a 6-exon, 2-promoter gene (Fig 4), and several H19 RNAs are produced via transcription from each promoter, including use of alternative transcription start sites, exon skipping, and intra-exonic alternative splicing. Analysis of GTEX has shown that most H19 transcripts are derived from promoter 2 [28, 37]. *H19* also has been found to be a 6-exon, 2-promoter gene in several non-human primates, including chimpanzee, gorilla, bonobo, orangutan, macaque, olive baboon, and marmoset, but not in the prosimian, mouse lemur, in which the gene appears to be poorly annotated in Ensembl, and DNA sequence similarity with human *H19* is limited to short stretches of several exons, unlike the other primates analyzed, in which all exons are very similar to their human analogues (94–100% identity [28, 37]). In other mammals *H19* appears to be a single-promoter gene with 5, 4 or 2 identifiable exons, depending on the species (Fig 4, Table 2). No *H19* gene could be found in 3 species (rabbit, opossum, platypus), either by sequence similarity searches with human or mouse *H19* DNA, by direct text-based searches of Ensembl or UCSC browsers, or by genomic mapping using species-specific H19 cDNAs (Table 2). For these species, poor quality of the genome sequences may be the major problem, as BLASTN searches using a corresponding H19 cDNA did not yield any identical or even similar gene segments.

**Table 2:** Percent nucleotide identity with human *H19* exons^¶^.

Species	Exon 1a (253 bp)	Exon 1 (1358 bp)	Exon 2 (135 bp)	Exon 3 (113 bp)	Exon 4 (123 bp)	Exon 5 (632 bp)
gorilla	98	99	96	98	100 (120)	98
olive baboon	94	96	91	96 (106)	94	96
cow	No match	87 (120)	No match#	No match#	89 (81)	92 (51)
pig	No match	85 (630)	87 (60)	No match	94 (67)	92 (79)
horse	No match	93 (430)	92 (86)	No match#	94 (63)	91 (186)
cat	No match	87 (530)	93 (56)	No match	90 (85)	89 (165)
dog	No match	89 (456)	97 (30)	No match#	94 (82)	97 (156)
mouse	No match	92 (354)	94 (35)	No match	95 (41)	92 (62)
rat	No match	91 (487)	94 (35)	No match	95 (85)	94 (81)
guinea pig	No match	90 (293)	No match	No match	No match	90 (101)
rabbit	No match	No match	No match	No match	No match	No match
elephant	No match	92 (318)	97 (31)	No match	94 (31)	96 (52)
armadillo	No match	90 (218)	No match	No match	No match	91 (113)
megabat	No match	93 (396)	94 (35)	No match	95 (66)	100 (23)
wallaby	No match	No match#	No match#	No match#	No match#	No match#
Tas devil	No match	No match#*	No match#*	No match#*	No match#*	No match#*
opossum	No match	No match	No match	No match	No match	No match
platypus	No match	No match	No match	No match	No match	No match

^¶^Number of base pairs aligned is in parenthesis if less than length of human exon.

*Poor DNA sequence quality

No match—no exon detected

^#^Exon is present in genome based on match with homologous or heterologous cDNA.

### *IGF2/Igf2* and *H19* gene expression

**Fig 5:**
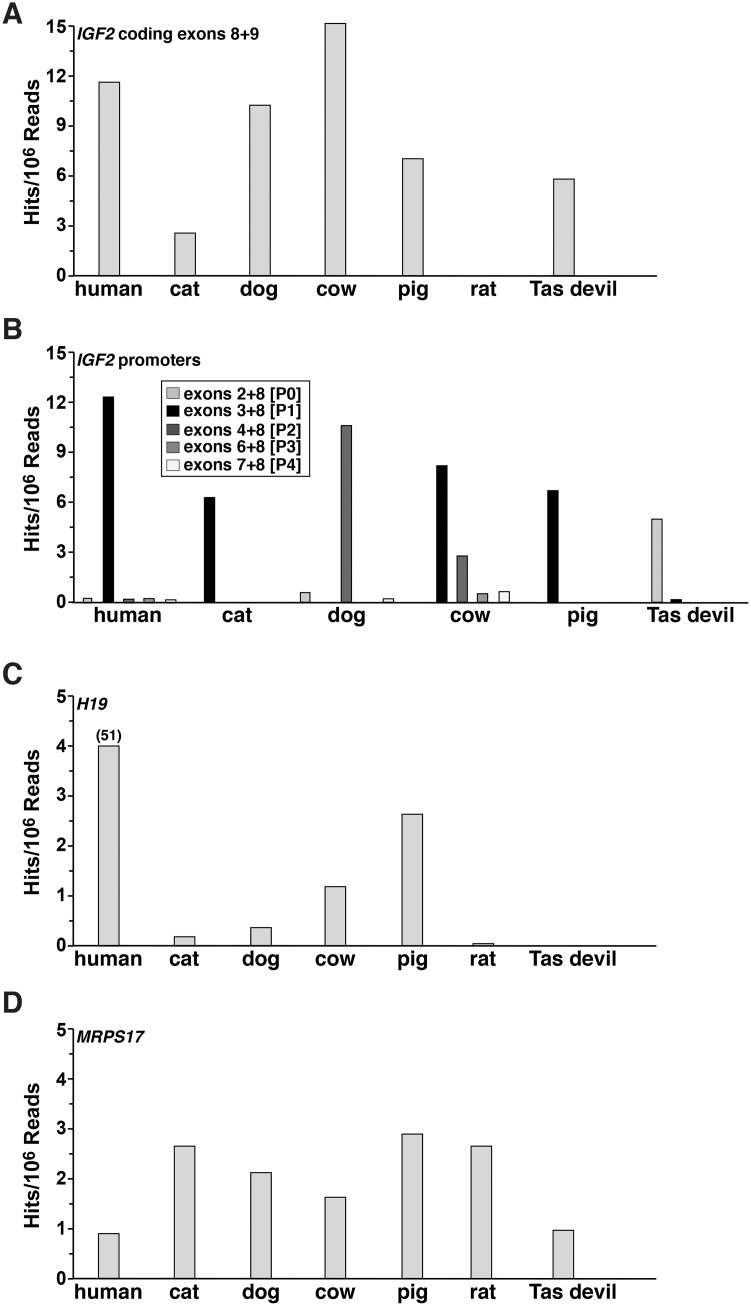
*IGF2/Igf2* and *H19* gene expression in mammals. Data on *IGF2/Igf2*, *H19*, and control gene *MRPS17*/*Mrps17* transcript expression in liver was obtained by screening RNA-sequencing libraries found in NCBI SRA (the libraries searched are listed in S1 Table, and the probes used in S2 Table). Results were graphed as hits identified per number of sequence reads in the library. **A**. *IGF2/Igf2* mRNA levels were measured in human, cat, cow, dog, pig, rat, and Tasmanian (Tas) devil using probes containing coding exons that were equivalent to human exons 8 and 9 (see S2 Table). **B**. *IGF2/Igf2* transcripts were assessed using probes containing each noncoding exon fused to the 5’ end of the first coding exon (the equivalent of human exon 8, see S2 Table). These results measure potential promoter use. **C**. *H19* gene expression was evaluated in the same species as in A. **D**. *MRPS17*/*Mrps17* (a potential control transcript) gene expression was assessed in the livers of the same species as in A.

Analysis of information in the SRA NCBI data resource revealed that *IGF2/Igf2* transcripts were expressed at varying levels in different mammals in adult liver (Fig 5A). In these studies, the RNA sequencing libraries chosen to be interrogated were prepared by a single research team, in order to minimize technical and other variables that might influence the quality and comparability of the data (S1 Table), and were screened with species-specific equivalents of human exons 8 and 9, the two most 5’coding exons. Further analyses used probes containing individual 5’ UTR exons linked to the most 5’ coding exon (the equivalent of human exon 8), in order to map promoter-specific hepatic transcripts, and these investigations revealed variability in apparent promoter usage. P1 predominated in 4 species (human, cat, cow, pig), while P2 was highest in dog, and P0 in Tasmanian devil, (Fig 5B), although the putative Tasmanian devil promoters and noncoding exons are not similar to those in human *IGF2* (Table 1).

Analysis of the same RNA-sequencing libraries showed that *H19* gene expression also appeared to vary in mammalian liver RNA. It was minimal in rat and absent in Tasmanian devil, and was substantial in human (Fig 5C). Transcript levels for a presumptively constitutively expressed control gene, *MRPS17*, varied over a 2.5-fold range (Fig 5D).

### IGF2 protein sequences in mammals

**Fig 6:**
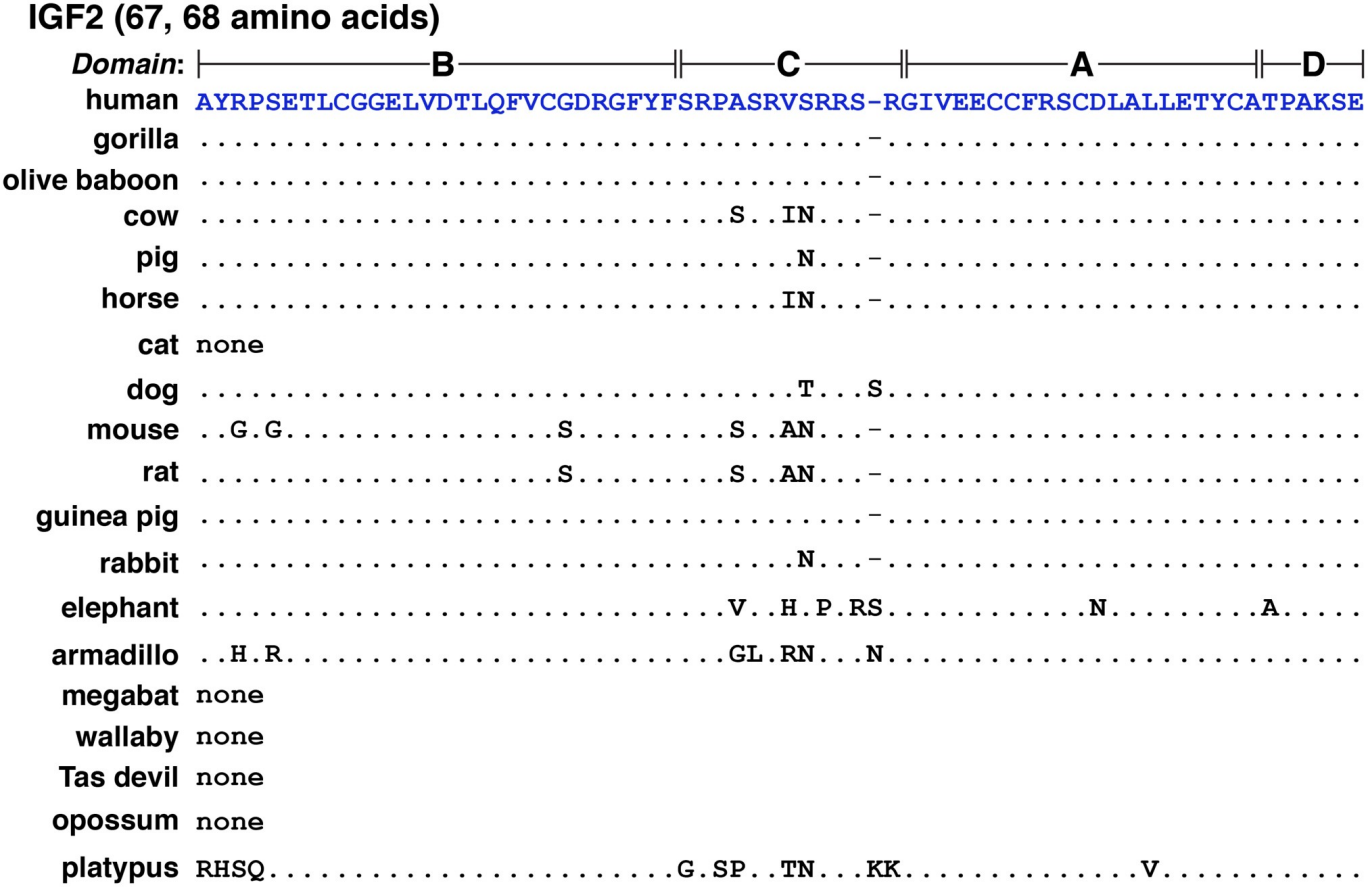
Alignments of mature IGF2. Amino acid sequences of IGF2 (67 or 68 amino acids) from different mammals are illustrated in single letter code. Dots depict identities, and differences among species are indicated. A dash depicts no residue. No IGF2 of this type could be identified in cat, megabat, wallaby, Tasmanian devil, or opossum, as indicated by the word ‘none’ (but see Fig 7).

**Fig 7:**
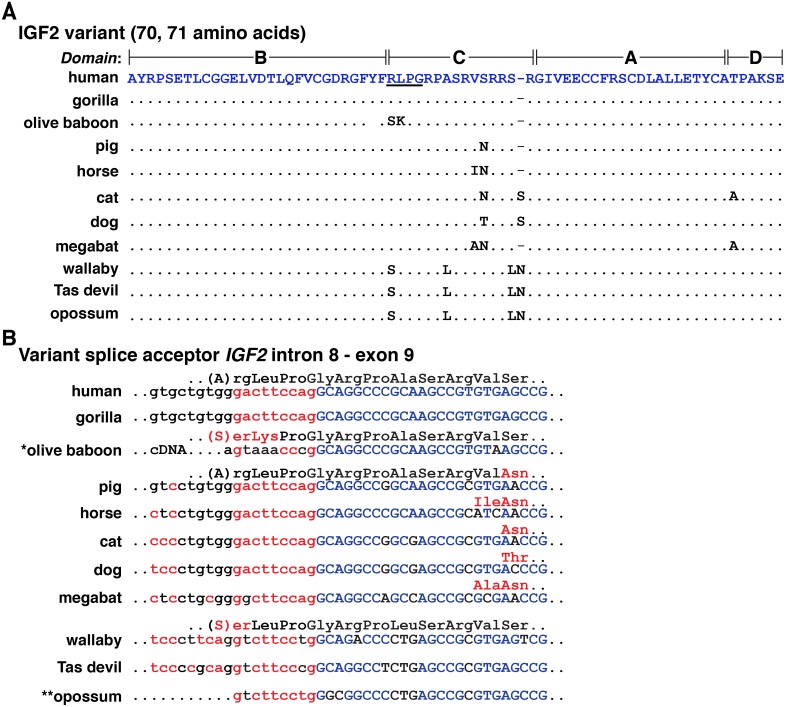
Mechanisms accounting for variant IGF2 proteins. **A**. Amino acid sequences of a mammalian IGF2 variants with 70 or 71 amino acids are depicted in single letter code. The additional residues are underlined for human IGF2. Dots depict identities, and differences among species are indicated. A dash depicts no residue. **B**. The molecular basis for 70- or 71-amino acid IGF2 is a consequence of alternative splicing into the equivalent of human *IGF2* exon 9, which adds an additional 9 nucleotides (in lower case and in red) to the 5’ end of the exon, and changes a serine codon into arginine-leucine-proline-glycine codons in *IGF2* transcripts in human, gorilla, and macaque. In olive baboon, as based on a cDNA sequence deposited in GenBank, a different 5’ end of exon 9 has been proposed, which results in predicted serine-lysine-proline-glycine codons. This sequence cannot be identified at the 3’ end of *IGF2* intron 8 in the olive baboon genome (as signified by *). In pig, horse, cat, dog, and megabat, different amino acids are found in the further COOH-terminal part of IGF2, as indicated in red. In wallaby and Tasmanian (Tas) devil, serine-leucine-proline-glycine comprise the variant amino acid quartet. This also may be true for opossum, but the relevant genomic DNA sequence is not available (thus **).

The 67-amino acid human IGF2 protein consists of 4 domains, termed B, C, A, and D (Fig 6) [45]. Mature human IGF2 is found within two types of protein precursors with different presumptive NH_2_-terminal signal peptides because of the inclusion or exclusion of exon 5 in *IGF2* mRNAs (Fig 2C). Among the 18 other mammals studied here, mature IGF2 appeared to be identical to the human protein in 3 species (gorilla, olive baboon, and guinea pig); there were single amino acid substitutions in pig and rabbit (Ser^36^ to Asn), and two changes in horse (Val^35^ to Ile, Ser^36^ to Asn) and dog (Ser^36^ to Thr, and an extra Ser after Ser^39^) (Fig 6, Table 3). In 4 other mammals, IGF2 was 68 amino acids in length (dog, elephant, armadillo, and platypus, Fig 6), and in 5 others, IGF2 consisted of 70 (megabat) or 71 residues (cat, wallaby, Tasmanian devil, and opossum; Fig 7, Table 3; and see below).

**Table 3:** Amino acid identities with human IGF2 (%).

Species	Signal peptide (24 AA)	Single peptide 2 (80 AA)	Mature IGF2 *(67 AA)	E Peptide (89 AA)
gorilla	100	100	100	98
olive baboon	100	none	100	94
cow	79	81	96	75
pig	75	none	99	85 (90 AA)
horse	80	29 (85 AA)	97	78 (90 AA)
cat	75 (26 AA)	86	96 (71 AA)	73 (64 AA)
dog	75 (26 AA)	65	97 (68 AA)	91 (90 AA)
mouse	80	none	91	82
rat	80	none	94	82
guinea pig	96	84	100	74 (90 AA)
rabbit	92	none	99	58 (90 AA)
elephant	75	66	90 (68 AA)	47 (83 AA)
armadillo	63 (28 AA)	none	91 (68 AA)	69
megabat	63	none	93 (70 AA)	82
wallaby	71	none	96 (71 AA)	65
Tas devil	71	none	96 (71 AA)	62
opossum	67	none	96 (71 AA)	58 (91 AA)
platypus	none	6 (88 AA)	90 (68 AA)	40 (83 AA)

*Several species have other versions of mature IGF2 (see Fig 7 and the text).

A variant 70-residue human IGF2 has been described, in which the amino acids Arg-Leu-Pro-Gly were predicted based on cDNA cloning and sequencing to replace Ser^29^ in the C-domain (Fig 7A) [46]. This protein was found in human serum [47], and upon experimental analysis, appeared to bind with lower affinity to the IGF1 receptor than did 67-amino acid IGF2 [47]. The mechanism responsible for this alternative human IGF2 is use of a variant upstream splice acceptor site that adds 9 nucleotides to the 5’ end of exon 9 in the resultant *IGF2* mRNA (Fig 7B). The same process appears to occur in *IGF2*/*Igf2* genes in gorilla, pig, horse, cat, dog, megabat, wallaby, and Tasmanian devil, leading to a 70- or 71-amino acid predicted protein (Fig 7B), and also accounts for the only IGF2 described in Uniprot for cat, megabat, wallaby, and Tasmanian devil (Fig 7A, Table 3), as well as for a second IGF2 in human, gorilla, pig, horse, and dog (Fig 7A). In olive baboon, a cDNA sequence in the NCBI nucleotide repository predicts a 70-amino acid variant IGF2, but the additional nucleotides 5’ to exon 9 (Fig 7B) differ from those found in its genome, so the existence of this larger protein cannot be validated yet. In opossum, a cDNA also is present in the NCBI nucleotide database that encodes a potential variant IGF2 (Fig 7B), but since no *Igf2* gene has been mapped to date in the opossum genome, this also remains unproven.

**Fig 8:**
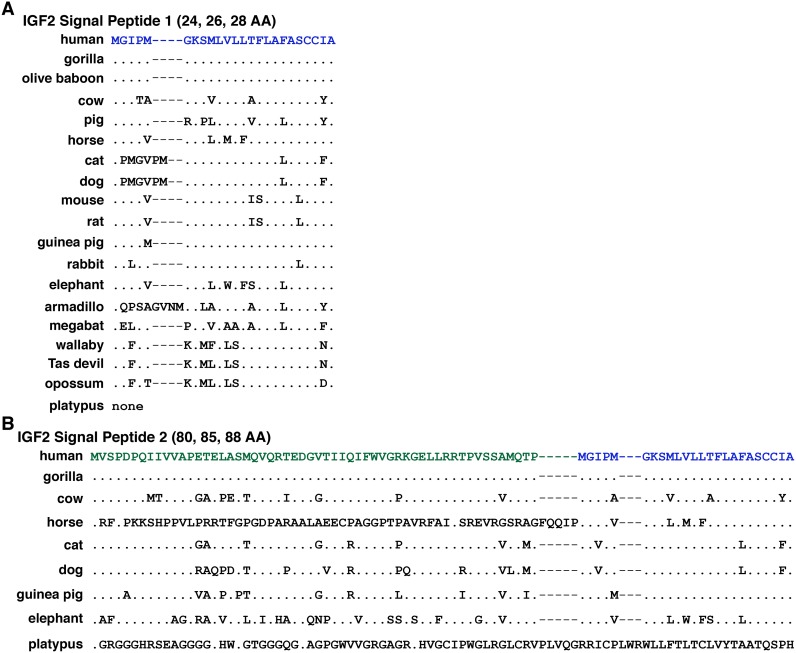
Alignments of IGF2 signal peptides. Amino acid sequences of IGF2 signal peptides from 19 mammals are shown in single letter code. **A**. 24-, 26-, or 28-residue IGF2 signal peptide 1 is found in all species except platypus. **B**. 80-, 85-, or 88-amino acid presumptive IGF2 signal peptide 2 can be detected only in human, gorilla, cow, horse, cat, dog, guinea pig, elephant, and platypus. The last 24 residues are identical to signal peptide 1 in human, gorilla, cow, horse, cat, dog, guinea pig, and elephant. For A and B, dots depict identities, dashes indicate no residues, and differences among species are shown. Note that in the platypus *Igf2* gene, only a larger signal peptide is predicted that is unrelated in amino acid sequence to other the species depicted.

There are two potential human IGF2 signal peptides, although the primary impetus for this statement is derived from the putative 236-amino acid IGF2 precursor protein being considered as a major product of the human *IGF2* gene in genome databases such as gnomAD (https://gnomad.broadinstitute.org; formerly termed ExAC [48, 49]). The more likely signal peptide has 24 amino acids and begins with a methionine codon near the 5’ end of *IGF2* exon 8; the other is predicted to have 80 residues, and is encoded by exons 5 (54 codons) and 8 (26 codons), with the last 24 residues being identical to those in the shorter signal peptide (Figs 2C and 8, Table 3), although there are no functional data to support the existence of the larger or of an internal signal sequence, and the transcript encoding this IGF2 precursor is minimally expressed in adult human tissues [37]. The smaller signal peptide can be detected in 17/18 of the other mammals analyzed (all but platypus), although its length is 26 amino acids in cat and dog, and 28 residues in armadillo. Only in gorilla and olive baboon is the 24-residue signal peptide identical to the corresponding part of the human IGF2 precursor (Fig 8A, Table 3). Based on genomic data, a peptide similar to the longer presumptive human IGF2 signal peptide of 80 amino acids is predicted in 8 other mammalian species, and corresponds to those mammals that have an analog of human *IGF2* exon 5 (Fig 3). However, no equivalent to exon 5 has been found platypus, and its predicted signal sequence is minimally related to the others (Fig 8B, Table 3). As noted above, there are no primary biochemical data demonstrating the existence of an IGF2 containing this potential 80-amino acid signal peptide, and it seems unlikely, as it is far longer than other described mammalian signal sequences [50, 51].

**Fig 9:**
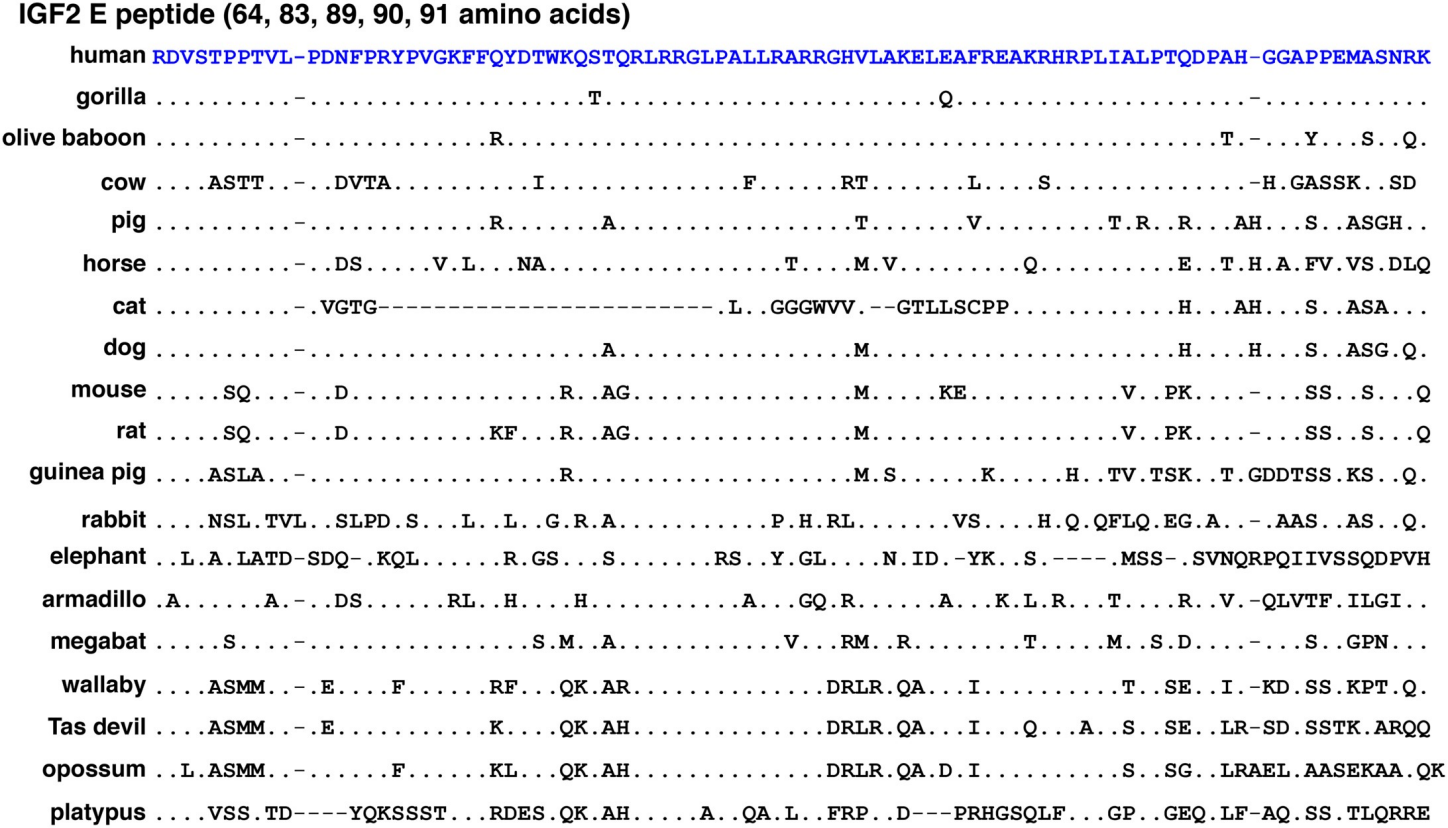
Alignments of IGF2 E peptides. Amino acid sequences of COOH-terminal IGF2 E peptides from 19 mammals are depicted in single letter code. Dots indicate identities, dashes depict no residues, and differences among species are shown. The IGF2 E domain comprises different lengths, ranging from 64 (cat) to 91 (opossum) amino acids.

The E peptide at the COOH-terminal end of the IGF2 protein progenitor consists of 89 amino acids in human and mouse (Fig 2C, Table 3). In other mammals it ranges in length from 64 residues (cat), to 83 (elephant, platypus), to 91 amino acids (opossum), with the majority containing 89 or 90 residues (Fig 9, Table 3). Although the E region is not well conserved, and was not identical in any two species of the 19 examined (Fig 9), it also has been identified in nonmammalian vertebrates, in which *Igf2* genes encode E domains ranging in length from 86 to 103 amino acids [52]. Potentially a reason for this variation among mammals and nonmammalian vertebrates is because of evolutionary drift of protein-coding segments of a gene that do not have fully specified functions [53].

### *IGF2-H19* locus organization in mammals

**Fig 10:**
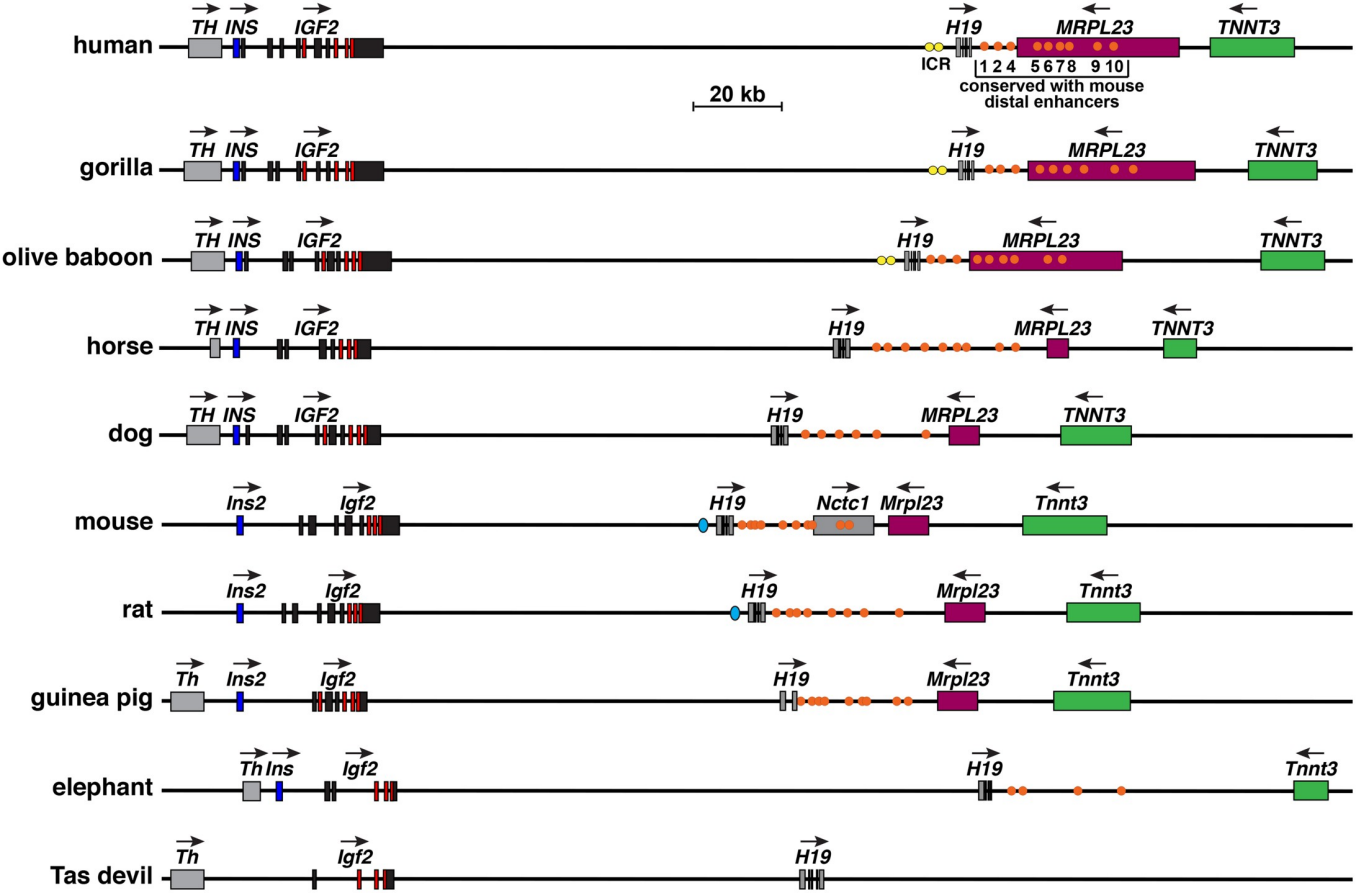
Comparison of *IGF2/Igf2*—*H19* locus and genes in mammals. Diagrams of human, gorilla, olive baboon, horse, and dog *IGF2—H19*, and mouse, rat, guinea pig, elephant, and Tasmanian (Tas) devil *Igf2—H19* genes and loci are shown. For *IGF2/Igf2* and *H19*, individual exons are indicated as boxes (coding regions are in red). Other genes are shown as single boxes, and include the following: tyrosine hydroxylase (*TH/Th*), insulin (*INS/Ins2*), noncoding RNA *Nctc1* (mouse only), mitochondrial ribosomal protein L23 (*MRPL23/Mrlp23*), troponin T3, fast skeletal type (*TNNT3/Tnnt3*). A horizontal arrow indicates the direction of transcription for each gene. Yellow (primate) or aqua ovals (mouse and rat) depict the imprinting control region (ICR) 5’ to *H19*, and orange circles indicate homologues of the 10 distal enhancers that were identified and functionally mapped in the mouse genome [40], and identified by DNA sequence similarity in the other genes (see Table 4). A scale bar is also shown. *Th* is not illustrated on the maps for mouse or rat, as it is separated from *Ins2* by ~226 Kb (mouse), and by ~222 Kb (rat).

The *IGF2*—*H19* locus is illustrated in Fig 10 for 10 different mammals in which the data are relatively complete. These loci exhibit several similarities in most of the species depicted. All contain *TH*/*Th*, *IGF2/Igf2*, and *H19* genes, although *Th* is located more than 220 kb from *Igf2* in both mouse and rat genomes (not shown). The genomes in most species pictured in Fig 10 also harbor *INS*/*Ins2*, *IGF2*/*Igf2*, *H19*, *MRPL23*/*Mrpl23*, and *TNNT3*/*Tnnt3* in the same linear order. However, *Ins* is absent in the sequenced Tasmanian devil genome, and was not identifiable by searching with the koala *Ins* DNA sequence (this likely represents a problem with genome quality). In addition, *Mrpl23* is absent in elephant, the length of *MRPL23*/*Mrpl23* or *TNNT3*/*Tnnt3* varies in several species, and their distance between each other or the distance from *H19* and *MRPL23* appears to be changed. Furthermore, in the mouse genome, *Nctc1* is present between *H19* and *Mrpl23* genes (Fig 10). More importantly, as determined by DNA sequence similarity with the human or mouse ICR, a recognizable ICR could be detected in only 5 species (human, gorilla, olive baboon, mouse, and rat) [54, 55]. Even though CTCF binding sites have been mapped 5’ to *H19* in wallaby [56, 57], they are sufficiently dissimilar to other species to not be recognizable in BLASTN searches with either human or mouse DNA segments. In contrast, we could identify putative enhancer elements 3’ to *H19* by DNA sequence similarity in locus maps from 9 of 10 species pictured in Fig 10, and at least one element was found in all mammals studied except for pig, rabbit, Tasmanian devil, opossum, and platypus (Fig 10, Table 4; some of these absences could be accounted for by low-quality genomic data in rabbit, platypus, and Tasmanian devil). To date, little is known about these enhancers beyond their functional characterization in transgenic mice [40–42], and the potential involvement of one of them in *Igf2* gene activation during skeletal muscle differentiation in tissue culture [58, 59]. Thus, their biological roles remain to be determined in most mammalian species. In opossum, analysis using the ECR browser revealed seventeen regions of similarity with the human *IGF2* –*H19* locus (> 65% identity for ≥ 100 base pairs) over ~340,000 Kb, but none of these were found near the putative enhancer segments or within the *Igf2* gene. Taken together, it is clear that the overall structure of this locus has undergone substantial modification during mammalian speciation, although aspects of the respective genes and their regulatory elements are identifiable in most of the mammals examined here.

**Table 4:** Percent nucleotide identity with mouse *Igf2-H19* locus enhancers^¶^.

Species	CS1 (218 bp)	CS2 (472 bp)	CS3* (214 bp)	CS4* (385 bp)	CS5 (385 bp)	CS6 (360 bp)	CS7 (231 bp)	CS8 (277 bp)	CS9 (486 bp)	CS10 (286 bp)
human	95 (76)	89 (251)	No match	84 (81)	86 (92)	87 (95)	95 (74)	85 (277)	84 (112)	93 (106)
gorilla	95 (75)	89 (251)	No match	84 (81)	86 (92)	87 (95)	91 (109)	87 (246)	85 (138)	91 (116)
olive baboon	95 (93)	88 (250)	No match	84 (81)	90 (41)	87 (95)	93 (108)	88 (246)	83 (138)	93 (116)
cow	No match	91 (103)	No match	84 (57)	89 (53)	89 (100)	89 (83)	95 (122)	88 (81)	88 (57)
pig	No match	No match	No match	No match	No match	No match	No match	No match	No match	No match
horse	No match	85 (233)	93 (41)	87 (122)	88 (90)	85 (160)	92 (87)	90 (248)	94 (108)	85 (191)
cat	93 (61)	83 (182)	No match	89 (104)	87 (92)	93 (45)	No match	87 (242)	No match	89 (126)
dog	No match	90 (88)	No match	90 (63)	92 (60)	89 (92)	No match	86 (275)	No match	90 (144)
rat	97	95	92	96	95	91	No match	99 (273)	94	No match
guinea pig	95 (122)	90 (251)	87 (159)	86 (106)	No match	90 (94)	93 (105)	87 (272)	84 (225)	94 (111)
rabbit	No match	No match	No match	No match	No match	No match	No match	No match	No match	No match
elephant	91 (95)	93 (51)	No match	No match	No match	No match	No match	87 (174)	No match	91 (89)
armadillo	No match	No match	No match	No match	No match	No match	No match	83 (93)	No match	No match
megabat	91 (117)	91 (124)	No match	84 (56)	86 (92)	93 (41)	89 (61)	86 (87)	87 (39)	89 (101)
wallaby	No match	No match	No match	No match	No match	No match	No match	83 (65)	No match	93 (55)
Tasmanian devil	No match	No match	No match	No match	No match	No match	No match	No match	No match	No match
opossum	No match	No match	No match	No match	No match	No match	No match	No match	No match	No match
platypus	No match	No match	No match	No match	No match	No match	No match	No match	No match	No match

^¶^Number of base pairs aligned is in parenthesis if less than length in mouse genome.

*Overlap with endodermal enhancers defined by Yoo-Warren et al (50).

No match—no DNA sequence identity detected

## Discussion

Human *IGF2* and mouse *Igf2* are complicated genes residing in a complex locus that encode a fairly simple single-chain secreted protein [13, 14, 21, 22, 37]. In both species, multiple gene promoters (5 for human, 4 for mouse) control the expression of several classes of *IGF2/Igf2* mRNAs that are translated into IGF2 protein precursors and ultimately processed into mature IGF2 (Fig 2). Activity of the *IGF2/Igf2* gene promoters in mice and humans is controlled by a number of developmental and tissue-specific mechanisms that have not been elucidated fully. Distal enhancers located 3’ to *H19* [40] may mediate some of these processes, and are in turn regulated by parental imprinting through DNA elements found 5’ to *H19* [16, 17, 55]. In most of the mammals studied here, a single-copy *IGF2/Igf2* gene has been identified that shares features with human *IGF2* and mouse *Igf2*, such as similarities in coding exons and in several noncoding exons (Fig 3 and Table 1). In most of these species, *IGF2/Igf2* resides within a locus that also contains *H19* and several other genes in identical order and orientation to those found in the human and mouse loci (Fig 10). The exceptions may be rabbit, opossum and platypus, in which no *H19* gene could be identified by similarity with human, mouse, or wallaby *H19* (Table 2), although this is likely to be secondary to poor DNA sequence quality in the respective genomes. The encoded IGF2 protein precursors also are similar, particularly in the mature segments of the molecule (Figs 6–9, Table 3). Moreover, in nearly all of the mammals studied here, the information annotated in genome repositories under-estimates the complexity of the overall structures of the respective *IGF2/Igf2* and *H19* genes, and in several species, the low quality of the genomic data precludes any conclusions about either gene.

Human *H19* is a 2-promoter, 6-exon gene (Figs 1 and 4) that uses alternative transcription start sites, exon skipping, and differential splicing within exons to generate multiple RNAs [28]. These mechanisms do not appear to be present in the non-primate mammalian species studied, in which only a single *H19* promoter has been identified in most (Fig 4, Table 2). Analysis of RNA-sequencing libraries showed that H19 RNA is expressed in adult liver in 6 of 7 different mammals examined here, but at varying levels (Fig 5), although these results should be considered preliminary, as library quality may be influenced by various factors including the input RNA and the steps or methods involved in library construction.

In mice and humans, parental imprinting is central to gene regulation for both *IGF2/Igf2* and *H19*, with an ICR located just 5’ to *H19* playing a key role in chromosome-of origin-specific gene activity through the actions of the CTCF transcription factor. As shown in mice, binding at the ICR in the maternal chromosome creates a boundary that prevents activation of *Igf2* [15–17]. In humans, rare individuals have been demonstrated to have presumptive inactivating deletions within the ICR, as they are associated with silencing of *H19* and bi-allelic expression of *IGF2* [55]. Few analogous studies have been performed in other mammals, and neither the human nor mouse ICR appear to be conserved among most of the species examined here, although of note CTCF binding sites have been detected 5’ to *H19* in wallaby, and the locus does appear to be reciprocally imprinted on allelic chromosomes [56]. Remarkably, homologues of putative distal enhancers functionally established and mapped 3’ to *H19* in the mouse *Igf2* –*H19* locus [40], and then identified in the human locus [37], also can be detected by DNA sequence similarity in corresponding locations in 12 of 17 other species (Table 4, Fig 10; in 3 species, rabbit, platypus, and Tasmanian devil, poor genome quality potentially contributes to this lack of identification).

Genetic, epigenetic, and environmental factors contribute to somatic growth in humans and other mammals [60, 61]. In humans, pediatric undergrowth and overgrowth disorders, such as Silver-Russell and Beckwith-Wiedemann syndromes, respectively, are associated with corresponding alterations in levels of IGF2 [7, 8], and changes in *IGF2/Igf2* gene expression influence tissue and organismal growth in pigs and mice [9–12]. An analogous growth-promoting role for IGF2 seems likely in other mammals, but experimental evidence is lacking to date. Similarly, as in humans, where every individual genome contains millions of DNA sequence polymorphisms [62, 63], other mammals also probably encode extensive DNA variation within their populations. This seems to be true in several nonhuman primates, including orangutans, where ~10 million SNPs have been identified recently [64], and in macaques, in which ~90 SNPs have been mapped near the *IGF2* gene [65] (also, see Mmul_8.0.1 at the following coordinates: chromosome 14: 1,954,752–1,963,881). As *IGF2* exhibits fairly extensive polymorphism in humans, with prevalent SNPs being found at the splice acceptor site between intron 4 and exon 5 (rs149483638; detected in ~2% of one large population [66]) and within the coding portion of exon 10 (rs61732764; changing R^156^ to H in the E domain in ~0.4% of humans in the same cohort [66]), modifications with the potential to alter *IGF2/Igf2* mRNA levels or change the protein sequence are likely to exist in additional mammals.

The important and multifactorial roles of IGF2 in growth, development, metabolic control, and other facets of human physiology and patho-physiology may be mirrored by its complex gene organization and patterns of regulation in diverse mammalian species. The organizational and DNA sequence congruence within the *IGF2/Igf2* –*H19* locus and the extensive amino acid similarity in the IGF2 protein among the mammalian species examined here suggest that constraining influences have maintained some essential common functional and regulatory mechanisms during mammalian speciation. Further study of other genes and loci involved in growth processes and related pathways using detailed analysis of information found in genomic and gene expression databases has the potential to add new insights regarding the origins of different physiological and pathological processes that affect humans and other mammals.

## Supporting information

S1 Table RNA-sequencing libraries screened for gene expression. (DOCX)

S2 Table Probes for screening RNA-sequencing libraries. (DOCX)
