# Supplementary material for: The insulin-like growth factor 2 gene in mammals: Organizational complexity within a conserved locus
Source: PLoS One. 2019 Jun 28;14(6):e0219155. doi: 10.1371/journal.pone.0219155 (PMC6599137; doi:10.1371/journal.pone.0219155)
Supplement: S1 Table — (DOCX) [file pone.0219155.s001.docx]

S1 Table: RNA-sequencing libraries screened for gene expression

| **Species** | **Organ** | **Experiment** | **Platform** | **Layout** | **Reads sequenced**  **(x 10^6^)** |
| --- | --- | --- | --- | --- | --- |
| Human | Liver | ERX1403333 | Illumina | paired | 58.1 |
| Pig | Liver | ERX1403346 | Illumina | paired | 46.7 |
| Cow | Liver | ERX1403338 | Illumina | paired | 37.4 |
| Rat | Liver | ERX1403323 | Illumina | paired | 53.1 |
| Cat | Liver | ERX1403298 | Illumina | paired | 79.5 |
| Dog | Liver | ERX1403293 | Illumina | paired | 36.4 |
| Tas devil | Liver | ERX1403331 | Illumina | paired | 34.9 |
