# Supplementary material for: The insulin-like growth factor 2 gene in mammals: Organizational complexity within a conserved locus
Source: PLoS One. 2019 Jun 28;14(6):e0219155. doi: 10.1371/journal.pone.0219155 (PMC6599137; doi:10.1371/journal.pone.0219155)
Supplement: S2 Table — (DOCX) [file pone.0219155.s002.docx]

S2 Table: Probes for screening RNA-sequencing libraries

| **Species** | **Gene** | **Probe** |
| --- | --- | --- |
| **Human** | *IGF2 ex 2+8* | TGCGGGGACTGCGCAGGGACTAGAGTACAGACACCAATGGGAATCCCAATGGGGAAGTCG |
|  | *IGF2 ex 3+8* | CAGCCTCAGCCCCAACTGCGAGGCAGAGAGACACCAATGGGAATCCCAATGGGGAAGTCG |
|  | *IGF2 ex 4+8* | GCGCCCGCGGACGCAGCCTCCAGCTTCGCGACACCAATGGGAATCCCAATGGGGAAGTCG |
|  | *IGF2 ex 5+8* | AGACGCACCCCGGTGAGCTCGGCCATGCAGACACCAATGGGAATCCCAATGGGGAAGTCG |
|  | *IGF2 ex 6+8* | GAGGTGGGCGGCAGCGTCGCCGGCTTCCAGACACCAATGGGAATCCCAATGGGGAAGTCG |
|  | *IGF2 ex 7+8* | TGCCCCAGCGAGCCTTCTGCTGAGCTGTAGACACCAATGGGAATCCCAATGGGGAAGTCG |
|  | *IGF2 ex 8+9* | TCGTCTGTGGGGACCGCGGCTTCTACTTCAGCAGGCCCGCAAGCCGTGTGAGCCGTCGCA |
|  | *IGF2 ex 8+9 var* | TCGTCTGTGGGGACCGCGGCTTCTACTTCAGACTTCCAGGCAGGCCCGCAAGCCGTGTGA |
|  | *H19 ex 2* | CCAGGTCTCCAGCTGGGGTGGACGTGCCCACCAGCTGCCGAAGGCCAAGACGCCAGGTCC |
|  | *H19 ex 4* | AAATGGTGCTACCCAGCTCAAGCCTGGGCCTTTGAATCCGGACACAAAACCCTCTAGCTT |
|  | *MRPS17 ex 3* | TATTTTAATAAGCGGAAAACCTACTTTGCTCACGATGCCCTTCAGCAGTGCACAGTTGGG |
|  |  |  |
| **Cat** | *IGF2 ex 2+8* | TTCGGGGACTACGCAGCGACTAGAGTACAGATGCCGATGGGGGTCCCAATGGGGAAGTCG |
|  | *IGF2 ex 3+8* | AGGCCTGTGGCCAGCCCCAGCCCCACAGAGATGCCGATGGGGGTCCCAATGGGGAAGTCG |
|  | *IGF2 ex 4+8* | AGCCTCCAGCCTCCAGCCTCCAGCCTTGCGATGCCGATGGGGGTCCCAATGGGGAAGTCG |
|  | *IGF2 ex 5+8* | none |
|  | *IGF2 ex 6+8* | none |
|  | *IGF2 ex 7+8* | AGCCCCAGCGAGCCTCCTGTCCAGCTGCAGATGCCGATGGGGGTCCCAATGGGGAAGTCG |
|  | *IGF2 ex 8+9* | TTGTCTGTGGGGACCGCGGCTTTTACTTCAGCCGCGTGAACCGCCGCAGCAGCCGTGGCA |
|  | *IGF2 ex 8+9 var* | TTGTCTGTGGGGACCGCGGCTTTTACTTCAGCAGGCCGGCGAGCCGCGTGAACCGCCGCA |
|  | *H19 ex 2* | CATTCTAGGCTGGCGCCGCGGGGACCCGGAACACCCAAGGGCTCGTTCCTCTGCGCTCCT |
|  | *H19 ex 4* | AGAAATGGTGCTACCCAGCTCACGCCTGGGCCTTGGAACTGGGACTTCTTCAAGTCCCCT |
|  | *MRPS17 ex 3* | TATTTTAATAAACGAAAAACCTACTTTGCTCATGATGCTCTTCAGCAGTGCACAGTTGGG |
|  |  |  |
| **Cow** | *IGF2 ex 2+8* | GTCCCTCCCTCACACGTCCCCCTCTGGGTGACATCAATGGGGATCACAGCAGGAAAGTCG |
|  | *IGF2 ex 3+8* | AATTGAGGCCCGTGGCCCCAGCCCGCAGAGACATCAATGGGGATCACAGCAGGAAAGTCG |
|  | *IGF2 ex 4+8* | CGGACGCAGCCTTCGGCCTCCAGCCTCGCGACATCAATGGGGATCACAGCAGGAAAGTCG |
|  | *IGF2 ex 5+8* | AGACGCACCCCGGTGAGCTCGGTCCTGCAGACATCAATGGGGATCACAGCAGGAAAGTCG |
|  | *IGF2 ex 6+8* | AAGGTGGGCGGCCCTGGTGCCGGCTTCCAGACATCAATGGGGATCACAGCAGGAAAGTCG |
|  | *IGF2 ex 7+8* | AGCCCCAGCGAGCCTCCTGTCCAGCTGCAGACATCAATGGGGATCACAGCAGGAAAGTCG |
|  | *IGF2 ex 8+9* | TTGTCTGTGGGGACCGCGGCTTCTACTTCAGCCGACCATCCAGCCGCATAAACCGACGCA |
|  | *IGF2 ex 8+9 var* | none |
|  | *H19 ex 2* | none |
|  | *H19 ex 4* | CAAGAATATGCTGCATTTTGGAACCACTACACACCCTAACTCAGGAATCAGCTCTGGAAG |
|  | *MRPS17 ex 3* | TATTTTAATAAGCGAAAAACCTACTTTGCCCACGATGCTCTCCAGCAGTGCACCGTTGGG |
|  |  |  |
| **Dog** | *IGF2 ex 2+8* | TTCGGGGACTACGCAGCGACTAGAGTACAGATGCCGATGGGGGTCCCGATGGGGAAGTCG |
|  | *IGF2 ex 3+8* | TGAGGTGAGCTGCTGCGGCCTGTGGCCCAGATGCCGATGGGGGTCCCGATGGGGAAGTCG |
|  | *IGF2 ex 4+8* | TCGGGGAGGCGCGCGCCCAGCGCGGGAGAGATGCCGATGGGGGTCCCGATGGGGAAGTCG |
|  | *IGF2 ex 5+8* | AGACGAAGGCCGGTGAGCTCGGTGTTGCAGATGCCGATGGGGGTCCCGATGGGGAAGTCG |
|  | *IGF2 ex 6+8* | GAGGTGGGCGGCAGCTGCGCCGGCTTCCAGATGCCGATGGGGGTCCCGATGGGGAAGTCG |
|  | *IGF2 ex 7+8* | AACCCCAGCGAGCCTCCTGTCCAGCTGCAGATGCCGATGGGGGTCCCGATGGGGAAGTCG |
|  | *IGF2 ex 8+9* | TTGTCTGTGGGGACCGCGGCTTCTACTTCAGCAGGCCGGCGAGCCGCGTGACCCGCCGCA |
|  | *IGF2 ex 8+9 var* | TTGTCTGTGGGGACCGCGGCTTCTACTTCAGACTTCCAGGCAGGCCGGCGAGCCGCGTGA |
|  | *H19 ex 2* | CTCCAGAGCCCGGGCCAAGGCGGGCCTCTGCGGGCGTCGACGGAGCAGGGATCGGTGCCT |
|  | *H19 ex 4* | AGAAATGGTGCTACCCAGCTCATGCCTGGGCCTTGGAAACCGGACTTCTTCAAGTCCTTC |
|  | *MRPS17 ex 3* | TATTTTAATAAGCGAAAAACCTACTTTGCTCATGATGCCCTTCAGCAGTGCACAGTTGGG |
|  |  |  |
| **Pig** | *IGF2 ex 2+8* | ACTACACAGCGACTAGAGGGCAGGTAACTGATTCCAATGGGGATCCCGATGAGGAAGCCG |
|  | *IGF2 ex 3+8* | TGAGGCGGGCAGCCCACCCAGCCCACAGCGATTCCAATGGGGATCCCGATGAGGAAGCCG |
|  | *IGF2 ex 4+8* | GGACGCAGCCTCCGGGCCTCCAGCCTTGCGATTCCAATGGGGATCCCGATGAGGAAGCCG |
|  | *IGF2 ex 5+8* | AGACGCGCCCCGGTGAGCTCGGTCCTGCAGATTCCAATGGGGATCCCGATGAGGAAGCCG |
|  | *IGF2 ex 6+8* | GAGGCGGGCGGCCCCTGCGCCGGCTTCCAGATTCCAATGGGGATCCCGATGAGGAAGCCG |
|  | *IGF2 ex 7+8* | CCCAGCGAGCCTCCTGTCCAGCTGCAGCAGATTCCAATGGGGATCCCGATGAGGAAGCCG |
|  | *IGF2 ex 8+9* | TTGTCTGCGGGGACCGCGGCTTCTACTTCAGCAGGCCGGCAAGCCGCGTGAACCGCCGCA |
|  | *IGF2 ex 8+9 var* | TTGTCTGCGGGGACCGCGGCTTCTACTTCAGACTTCCAGGCAGGCCGGCAAGCCGCGTGA |
|  | *H19 ex 2* | GGACATGACACAGTCCGGTGTGACGGAGAGGGACAGACGTGACGCCGTCCGGCCTTCCTG |
|  | *H19 ex 4* | ATGGTGCTACCCAGCTCATGCCTGGGCCTTGGACCCGGACTTCTTCAAGTCCTCCTAGCT |
|  | *MRPS17 ex 3* | TATTTTAATAAGCGAAAAACCTACTTTGCCCACGATGCTCTTCAGCAGTGCACCGTCGGG |
|  |  |  |
| **Rat** | *Igf2 ex 2+6* | CCTCCCACCAGCCCCAGTCCTTCCTCAGAGGTACCAATGGGGATCCCAGTGGGGAAGTCG |
|  | *Igf2 ex 3+6* | GCGCCAGCGGACCCGACCTTCGGCCTTGCGGTACCAATGGGGATCCCAGTGGGGAAGTCG |
|  | *Igf2 ex 4+6* | GAGATGGGCGGCAGCGTCGCCGGCTTCCAGGTACCAATGGGGATCCCAGTGGGGAAGTCG |
|  | *Igf2 ex 5+6* | AGCCCCAGCGGCCTCCTTATCCAACTTCAGGTACCAATGGGGATCCCAGTGGGGAAGTCG |
|  | *Igf2 ex 6+7* | TTGTCTGTTCGGACCGCGGCTTCTACTTCAGCAGGCCTTCAAGCCGTGCCAACCGTCGCA |
|  | *Igf2 ex 6+7 var* | none |
|  | *H19 ex 2* | GACATGACATGGTCCGGTGTGATGGAGAGGACAGAAGGACAGTCATCCAGCCTTCCTGGT |
|  | *H19 ex 4* | AAATGGTGCTACCCAGCTCATGTCTGGGCCTTTGAATCCGGGGACTTCTTTAAGTCCGTC |
|  | *Mrps17 ex 3* | TACTTTAATAAGCGGAAAACCTACTTTGCTCATGACGCCCTTCAGCAGTGCAGCGTCGGG |
|  |  |  |
| **Tas devil** | *IGF2 ex 2+8* | GAAAGAGGAGCCGCAGGACGGCGTCCCGAGATGGGTTTCCCAATGAAGAAGATGTTGCTG |
|  | *IGF2 ex 3+8* | CTCCGCGCCGCGCCCTTCGCAAAATCTGGGATGGGTTTCCCAATGAAGAAGATGTTGCTG |
|  | *IGF2 ex 4+8* | none |
|  | *IGF2 ex 5+8* | none |
|  | *IGF2 ex 6+8* | none |
|  | *IGF2 ex 7+8* | none |
|  | *IGF2 ex 8+9* | none |
|  | *IGF2 ex 8+9 var* | TTGTGTGTGGCGACCGCGGCTTCTACTTCAGTCTTCCCGGCAGGCCTCTGAGCCGCGTGA |
|  | *H19 ex 1* | CCGCCACCTTCTCCAGGAACTCTTTGCAGCCCGGGAGCCGGAGCGGAGAAAGCAAACAGT |
|  | *H19 ex 4* | none |
